# Mitral valve replacement in mitral stenosis; the problem of small left ventricle

**DOI:** 10.1186/s13019-020-01108-z

**Published:** 2020-04-22

**Authors:** Hesham Alkady, Ahmed Saber, Sobhy Abouramadan, Ahmed Elnaggar, Sherif Nasr, Eman Mahmoud

**Affiliations:** 1 Department of cardiothoracic surgery, Cairo University, Kasralaini str., Almanial, Cairo, Egypt; 2 Department of cardiothoracic surgery, Fayoum University, Fayoum, Egypt; 3 Department of cardiology, Fayoum University, Fayoum, Egypt

**Keywords:** Mitral stenosis, Mitral valve replacement, Small left ventricular cavity, Heart failure, Low cardiac output syndrome

## Abstract

**Background:**

Mitral valve stenosis in adults especially due to rheumatic heart disease may be associated with a smaller than normal left ventricular cavity. Mitral valve replacement in such cases may lead to hemodynamic instability either during weaning from cardiopulmonary bypass or in the early postoperative period manifested by the need for inotropic support and even mortality due to low cardiac output syndrome.

**Patients and methods:**

184 patients with predominately severe stenotic mitral valves who underwent elective isolated mitral valve replacement in the period between January 2012 and January 2018 at our hospital were included in this study. Patients were divided into 2 matched groups; (small LV group) consisting of 86 cases and (normal or dilated LV group) consisting of 98 cases.

**Results:**

There were no statistically significant differences in operative details among both groups apart from the need for inotropic support and intra-aortic balloon pump due to low cardiac output which were statistically significantly higher in (small LV group) than (normal or dilated LV group) with a *p*-values of 0.01 and 0.03 respectively. Within the ICU stay only the incidence of occurrence of heart failure was significantly higher in (small LV group) with a *p*-value of 0.008. No statistically significant difference could be elicited in the in-hospital mortality between both groups (*p*-value = 0.1).

**Conclusion:**

Patients with mitral valve stenosis and small left ventricular cavity are in a higher need for inotropic and even mechanical support after mitral valve replacement as well as at a higher risk for the development of heart failure before hospital discharge than patients with mitral stenosis and normal-sized left ventricular cavity.

## Background

Mitral valve stenosis (MS) in adults especially due to rheumatic heart disease may be associated with a smaller than normal left ventricular cavity. This might be attributed to inadequate left ventricular preloading or chronic myocardial damage due to rheumatic heart disease resulting in left ventricular atrophy [1]. Sudden increase of blood inflow from the left atrium to the small “unprepared” left ventricle after mitral valve replacement (MVR) in such cases may lead to hemodynamic instability either during weaning from cardiopulmonary bypass (CPB) or in the early postoperative period manifested by the need for inotropic support and even mortality due to low cardiac output (COP) syndrome [2]. In this study we share our experience with the outcomes of mitral valve replacement in patients with long-standing severe mitral stenosis and small left ventricular cavity.

## Patients and methods

In this study the data of 86 consecutive patients with predominately severe stenotic mitral valves (mitral regurgitation ≤ + 2) and small left ventricular cavity (determined by the lower reference values from the NORRE study according to age, gender and body surface area) [3] who underwent elective isolated MVR in the period between January 2012 and January 2018 at our hospital were retrospectively collected and analyzed (small LV group). Another group of 98 patients with predominately severe stenotic mitral valves but with normal-sized or dilated left ventricles who underwent elective isolated MVR over the same period were matched to (small LV group) using propensity scores according to preoperative patient characteristics (normal or dilated LV group). All these cases were not amenable to balloon mitral valvoplasty either due to excessive calcification at the leaflet tips or the presence of left atrial thrombus. Cases associated with any other significant valvular or coronary artery diseases necessitating surgical intervention were not included. In addition, patients with impaired left ventricular function (ejection fraction ≤50) were excluded. Our study was approved by the institutional ethical board and because of its retrospective nature, patient consent was waived. All patients received preoperative transthoracic 2D-echocardiography to evaluate the mitral as well as other cardiac valves and all cardiac chambers. Coronary angiography was done for patients aged >45 years preoperatively to delineate any coronary artery disease. Severe MS necessitating surgery was defined by a mitral valve area (MVA) of < 1.0 cm^2^ and a mean pressure gradient across the mitral valve of > 10 mmHg. Grading of associated mitral regurgitation (MR) was carried on according to jet area and Vena contracta [4]. The size of the left ventricular cavity was assessed through echocardiography using the Simpson’s biplane method [5]. Preoperative, operative and postoperative data were obtained from hospital records and were compared for both groups regarding; the size and type of implanted prosthesis, intra-operative time parameters (aortic cross-clamp, CPB and total operative times), need for inotropic support (Epinephrine infusion) or intra-aortic balloon counter-pulsation (IABP) during weaning from CPB to maintain mean arterial blood pressure > 90mHg, duration of mechanical ventilation and ICU stay, occurrence of postoperative complications (heart failure, renal impairment, cerebro-vascular stroke and bleeding), in-hospital mortality and total hospital stay. Heart failure was defined as dyspnea or impaired arterial blood gases in association with radiographic signs of pulmonary congestion. Renal impairment was defined as an increase in the serum creatinine to > 2 mg/dl or twice the level of preoperative creatinine. Patients were followed up through outpatient clinics and telephone calls regarding late occurrence of heart failure and cardiac mortality.

### Statistical analysis

Data were coded and then entered into the SPSS statistical package (Statistical Package for the Social Sciences) version 22. Quantitative data were summarized using the mean ± standard deviation, while categorical data were presented as frequency (count) and relative frequency (percentage). The means of continuous variables were compared between groups by Student’s t-test while rates and proportions were compared by the Chi Square or Fisher exact tests. The propensity score matching was obtained by multiple logistic regression, based on preoperative patient characteristics (Table 1). A *p*-value < 0.05 was considered as significant. Kaplan-Meier curves were used to determine survival and freedom from heart failure.

**Table 1:** Preoperative patient characteristics. AF; atrial fibrillation, CVS; cerebrovascular stroke, COPD; chronic obstructive pulmonary disease, CRF; chronic renal failure, LVEDD; left ventricular end-diastolic diameter, LVESD; left ventricular end-systolic diameter, LVEDV; left ventricular end-diastolic volume, PG; pressure gradient

Preoperative patient characteristics	Small LV group (*N* = 86)	Normal or dilated LV group (*N* = 98)	*P*-value
**Age (years)**	34 ± 7	35 ± 8	0.02
**Gender**			
**Males/Females**	36 (42%)/ 50 (58%)	46 (47%)/ 52 (53%)	0.49
**Body surface area (m**^**2**^**)**	1.68 ± 0.12	1.71 ± 0.16	0.15
**Chronic AF**	58 (67%)	68 (69%)	0.78
**Previous CVS**	18 (21%)	25 (25.5%)	0.46
**Previous balloon commisurotomy**	24 (28%)	37 (38%)	0.16
**Co-morbidities**			
Diabetes mellitus	6 (7%)	8 (8%)	0.76
Systemic hypertension	5 (5.8%)	3 (3%)	0.36
COPD	2 (2%)	2 (2%)	0.89
Residual hemiparesis after previous CVS	4 (4.6%)	6 (6.1%)	0.66
CRF on regular dialysis	1 (1.16%)	–	0.46
**Preoperative Echocardiographic date**			
Mean LVEDD (cm)	4.94 ± 0.80	5.51 ± 1.70	**0.002**
Mean LVESD (cm)	3.35 ± 0.18	3.54 ± 0.30	**0.001**
Mean LVEDV index (ml/m^2^)	34.48 ± 2.7	65.67 ± 3.4	**0.001**
Mean mitral valve area (cm^2^)	0.61 ± 0.20	0.62 ± 0.60	0.14
Mean PG across the mitral valve (mmHg)	20.68 ± 2.56	20.03 ± 2.36	0.08
Degree of associated mitral regurgitation			**0.001**
≤ + 1	75 (87%)	8 (8%)	
≤ + 2	11 (13%)	90 (92%)	
Mean left atrial diameter (cm)	5.12 ± 0.58	5.28 ± 0.61	0.07
Mean pulmonary artery pressure (mmHg)	54.17 ± 9.08	51.78 ± 9.60	0.08
Evidence of left atrial thrombus	5	7	0.72

## Results

### Preoperative characteristics

All patients were in New York Heart Association (NYHA) class III preoperatively. Table 1 summarizes preoperative epidemiological, clinical and echocardiographical data. There were no statistically significant differences between both groups regarding preoperative patient characteristics apart from left ventricular end-diastolic and end-systolic diameters as well as end-diastolic volume index. In addition, (normal or dilated LV group) had significantly higher degrees of associated mitral regurgitation (MR) than (small LV group) with a *p*-value of 0.01.

### Operative data

In (small LV group) 36 patients (42%) were operated upon minimally invasive through right anterolateral minithoracotomy in the 5th intercostal space, 15 patients (17%) through lower ministernotomy till the 4th intercostal space and the rest (35 patients) were operated upon conventionally through full median sternotomy. On the other hand, in (normal or dilated LV group) 44 patients (45%) were operated upon through right anterolateral minithoracotomy, 18 patients (18%) through lower ministernotomy and the rest (36 patients) were operated upon through full median sternotomy. These different techniques are attributed to our program in minimally invasive mitral valve surgery which was established later in the study in 2014 and was started with the ministernotomy incision and then shifted to the right minithoracotomy incision in 2015. The pathology of mitral valve stenosis was rheumatic in origin in both groups except for 2 cases (2%) in (small LV group) and 3 patients (3%) in (normal or dilated LV group), where stenosis was attributed to degenerative changes. Significant mitral annular calcification (MAC) was not encountered in our study patients [6]. The mitral valve was replaced through left atriotomy in full sternotomy as well as right minithoracotomy cases and was replaced through the transseptal approach in lower ministernotomy cases. Myocardial protection was done in all patients through ante-grade cold blood cardioplegia via the aortic root as well as moderate hypothermia (rectal temperature, 28 °C). In all cases preservation of the posterior mitral leaflet could be done. All patients received St. Jude mechanical mitral prostheses (SJM; St. Jude Medical Inc.; Minneapolis, Minn). Table 2 shows the different techniques used for MVR, the sizes of used mechanical prostheses, intra-operative time parameters as well as need for inotropic support and IABP in both groups. There were no statistically significant differences in operative details among both groups apart from the need for inotropic support and IABP due to early low COP (immediately after weaning from CBP) which was statistically significantly higher in (small LV group) than (normal or dilated LV group) with a *p*-values of 0.01 and 0.03 respectively.

**Table 2:** Operative data. MS; mitral stenosis, MVR; mitral valve replacement, CPB; cardiopulmonary bypass, IABP; intra-aortic balloon pump, COP; cardiac output

Operative data	Small LV group (*N* = 86)	Normal or dilated LV group (*N* = 98)	*P*-value
**Pathology of MS**			0.76
Rheumatic	84 (98%)	95 (97%)	
Degenerative	2 (2%)	3 (3%)	
**Techniques of MVR**			0.50
Full median sternotomy	35 (41%)	36 (37%)	
Lower ministernotomy	15 (17%)	18 (18%)	
Right anterolateral minithoracotomy	36 (42%)	44 (45%)	
**Sizes of mitral prostheses (mm)**			0.50
25	2	1	
27	80	89	
29	4	8	
**Intra-operative time parameters (min)**			
Total operative time	219.25 ± 19.86	213.70 ± 22.12	0.07
CPB time	85.67 ± 7.72	83.43 ± 8.06	0.06
Cross-clamp time	67.80 ± 10.23	69.37 ± 9.21	0.27
**Need for inotropic support**	80 (93%)	60 (45%)	**0.01**
**Need for IABP due to low COP syndrome**	6 (7%)	1 (1%)	**0.03**

### Postoperative results

Table 3 shows the postoperative outcomes. There were no statistically significant differences between both groups in concern of postoperative complications apart from the occurrence of heart failure within the ICU stay (*p*-value = 0.008) which was significantly higher in (small LV group). These cases were managed by increasing the doses of inotropes as well as vigorous diuresis. There were 3 in-hospital mortalities in (small LV group) due to refractory low COP syndrome despite maximal inotropic support and IABP. On the other hand no in-hospital mortality occurred in (normal or dilated LV group). However despite this difference in the in-hospital mortality between both groups, no statistical significance could be elicited (*p*-value = 0.10).

**Table 3:** Postoperative results

Postoperative results	Small LV group (*N* = 86)	Normal or dilated LV group (*N* = 98)	*P*-value
**Duration of mechanical ventilation (hours)**	6.46 ± 1.38	6.08 ± 1.36	0.06
**Duration of ICU stay (days)**	3.76 ± 1.05	3.48 ± 1.08	0.07
**Total hospital stay (days)**	8.87 ± 1.18	8.59 ± 0.90	0.07
**Postoperative complications**			
Renal failure	2 (2.5%)	3 (3%)	0.75
Cerebrovascular stroke	–	1 (1%)	0.53
Heart failure	10 (11.6%)	2 (2%)	**0.008**
Re-exploration for bleeding	1 (1.16%)	–	0.47
**In-hospital mortality**	3 (3.4%)	–	0.10

**Fig. 1:**
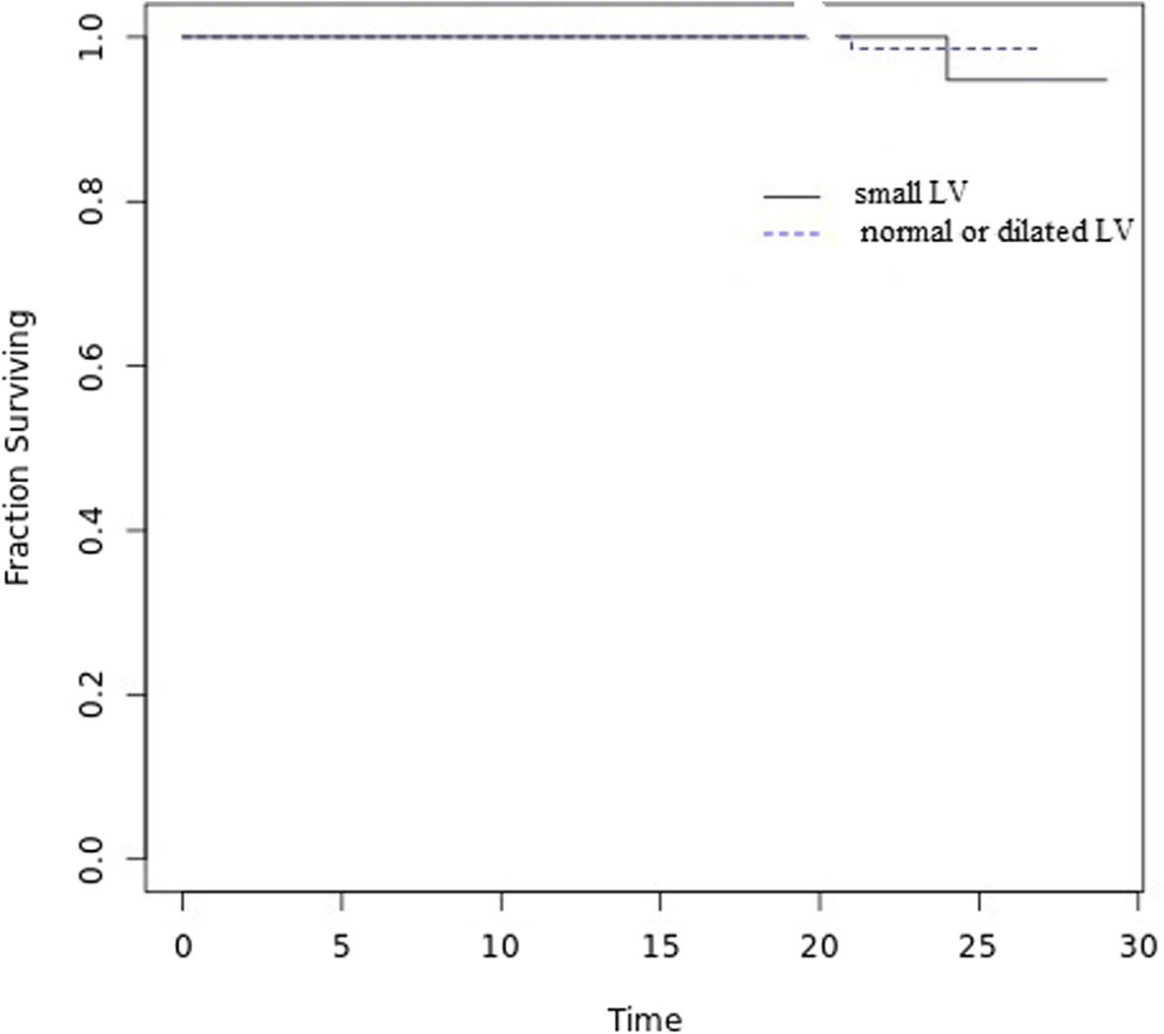
Kaplan-Meier curve showing survival in both groups during the follow-up period

**Fig. 2:**
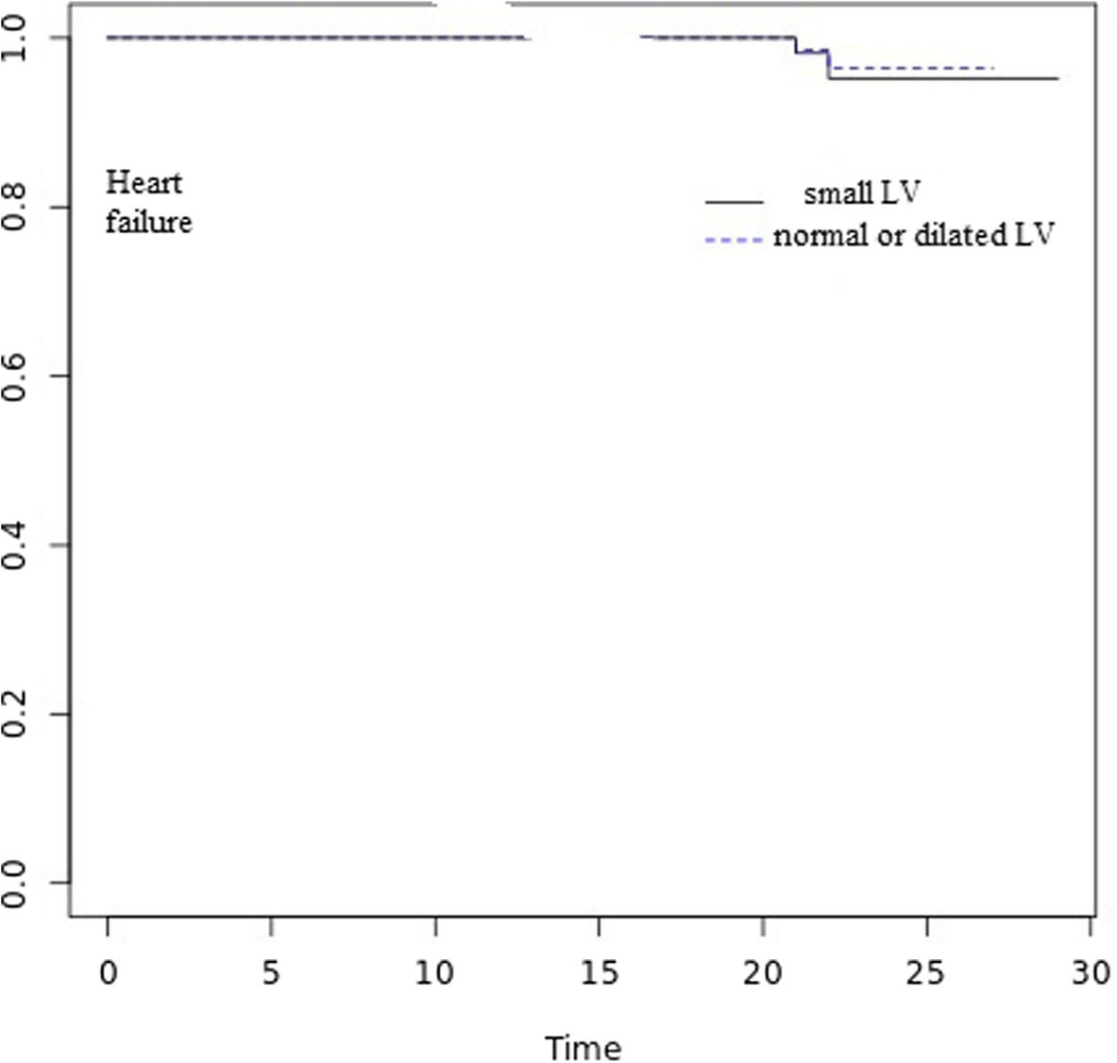
Kaplan-Meier curve showing the occurrence of heart failure in both groups during the follow-up period

96.5% of patients in (small LV group) and 94.9% of patients in (normal or dilated LV group) could be followed up for a mean period of 2 ± 1.2 years after discharge. There was no statistically significant difference between both groups during the follow up period in mortality; one in (small LV group) due to endocarditis after 8 months and one in (normal or dilated LV group) after 16 months due to massive valve thrombosis or in re-admission due to heart failure; two patients in both groups after 3 and 4 months respectively. Figs 1 and 2

## Discussion

There is little information in the literature regarding the physiologic and structural effects of mitral stenosis, especially of rheumatic origin, on the left ventricle and its size [7, 8]. However, in our practice, the association of predominately severe mitral stenosis and small left ventricular cavity is not uncommon especially when the degree of associated mitral regurgitation is minimal. This was obvious in our study cohort where patients in (normal or dilated LV group) had significantly higher degrees of associated mitral regurgitation. The increase in the size of the left ventricular cavity in this group may be attributed to the higher preload conditioning of the left ventricle from associated mitral regurgitation. Also the pathology of mitral stenosis in our community is still mainly due to rheumatic heart disease rather than degenerative processes.

Repair of stenotic mitral valves especially due to rheumatic pathology, although remains better than replacement, is not easy in many cases and the surgeon may be forced to replace the valve if repair is considered impossible or non-durable [9, 10]. Mitral valve replacement in chronic mitral valve stenosis with small left ventricular cavity especially due to rheumatic cause may be associated with systolic left ventricular dysfunction after weaning from cardiopulmonary bypass and/ or early postoperative period. This is in part due to the sudden volume overload of the small left ventricle not accustomed to the increased blood influx from the left atrium as well as due to partial or even complete excision of one or both mitral leaflets and subvalvular apparatus during mitral valve replacement [11]. Although no doubt exists regarding importance of the mitral valve apparatus including the chordea tendinea and papillary muscles to left ventricular contractility and every effort is to be made to preserve as much as possible of the mitral valve during replacement [12, 13]. However this might be difficult in mitral stenosis due to the need for placing a prosthesis with an adequate size to avoid patient prosthesis mismatch (PPM) which is defined as placing a small prosthesis in relation to the body surface area with an effective orifice area (EOA) index ≤1.2 cm2/m2 [14, 15]. Such PPM is proved to be associated with a residual pressure gradient across the mitral valve resulting in the persistence of pulmonary hypertension and recurrence or increase of associated functional tricuspid regurgitation [16, 17]. Therefore we routinely preserve the posterior mitral leaflet during mitral valve replacement in mitral stenosis to ameliorate the postoperative left ventricular systolic dysfunction. In addition we do not support at all the idea of placing a small-sized prosthetic valve like 25 mm in such patients as an attempt to avoid the early left ventricular systolic dysfunction after MVR [2]. We always try to place the largest possible prosthetic valve for fear of PPM on the long run.

According to our study patients with MS and small left ventricle needed more inotropic support and IABP during weaning off CBP after MVR to maintain a mean arterial blood pressure >90 mmHg (*P* value = 0.01 and 0.03 respectively) and showed higher incidence of heart failure before discharge from ICU (*p*-value = 0.008). This is why in such patients we tend to use a reasonable dose of inotropes (e.g. Epinephrine at a rate of 50 ng/kg per minute as a continuous infusion) almost routinely during weaning off CBP and withdraw this inotropic support very gradually on the first postoperative day. In addition intravenous volume intake should be carefully administered both during weaning off CBP and during the ICU stay.

Finally our study has some limitations in the form of the retrospective nature and single-center experience. In addition, no significantly higher mortality could be elicited both during the primary hospital stay or the follow up period in patients with mitral stenosis and small left ventricle undergoing MVR. Therefore more future studies with larger cohort may be needed.

## Conclusion

Patients with mitral valve stenosis and small left ventricular cavity are in a higher need for inotropic and even mechanical support after mitral valve replacement as well as at a higher risk for the development of heart failure before hospital discharge than patients with mitral stenosis and normal-sized left ventricular cavity. Therefore inotropic support should be used expediently after weaning from cardiopulmonary bypass and withdrawn carefully during early postoperative period after MVR in such patients.

## Data Availability

The datasets used and/or analysed during the current study are available from the corresponding author on reasonable request.

## Abbreviations

COP: Cardiac output syndrome
CPB: Cardiopulmonary bypass
EOA: Effective orifice area
IABP: Intra-aortic balloon counter-pulsation
LV: Left ventricle
MAC: Mitral annular calcification
MR: Mitral regurgitation
MS: Mitral stenosis
MVA: Mitral valve area
MVR: Mitral valve replacement
NYHA: New York Heart Association
PPM: Patient prosthesis mismatch
